# Environmental surfaces used in entry-day corralling likely contribute to the spread of influenza A virus in swine at agricultural fairs

**DOI:** 10.1038/emi.2016.138

**Published:** 2017-02-22

**Authors:** Sarah E Lauterbach, Michele M Zentkovich, Sarah W Nelson, Jacqueline M Nolting, Andrew S Bowman

**Affiliations:** 1 Department of Veterinary Preventive Medicine, The Ohio State University, 1920 Coffey Road, Columbus, OH 43210, USA

**Dear Editor,**

Swine are considered an important host of influenza A virus (IAV), allowing for rapid reassortment that can produce novel viruses leading to human infections resulting in outbreaks and pandemics.^1^ Therefore, zoonotic transmission of IAV creates a major public health threat. A small portion of the United States swine population (~1.5%) is raised for youth education in small farm settings and exhibited at agricultural fairs, which encourages increased human-animal interaction, creating an important interface for zoonotic IAV transmission (Bliss *et al*,^2^ *Journal of the American Veterinary Medical Association*, in press). However, being a small niche, exhibition swine are often overlooked as active participants in disease transmission and pathogen dissemination.^3^ Nevertheless, most documented cases of swine-to-human IAV transmission have been associated with exposure to swine during agricultural fairs.^4, 5^ In 2012, 309 confirmed cases of H3N2 variant infection were reported, with >90% of individuals infected that year reporting swine contact at agricultural fairs.^6^

Active surveillance has revealed that when IAV is detected in swine at an agricultural fair, by the last day of the fair >60% of swine may be infected.^7^ Conversely, it has been estimated that 1.5% of pigs arrive at fairs with active infection.^2^ These point-in-time estimates illustrate the rapid IAV transmission that is occurring in swine at agricultural fairs, which appears faster than predicted by a typical direct contact transmission model.^8^ This led us to investigate potential underlying mechanisms that can enhance viral spread in agricultural fairs. Identifying routes of IAV transmission in these settings will allow animal health officials to focus efforts on developing control strategies that will likely limit viral spread and lessen the public health risk.

Individual pigs are typically weighed and identified upon arrival at agricultural fairs to ensure they meet show standards. This corralling commonly occurs at a central location within the barn and is accomplished by moving pigs single-file through a chute (Figure 1A). Previous testing has identified IAV contamination on environmental surfaces in live animal markets,^9^ leading Bliss *et al* to hypothesize that entry-day corralling may contribute to viral transmission during fairs via viral contamination of chute surfaces.^2^ The present study investigated IAV contamination of chute surfaces during corralling activities.

Prior to corralling, three swine-contact surfaces in the chute were identified for sampling (A, B, C) at six agricultural fairs, identified as Fairs 1–6. Each surface was wiped with cotton gauze (Convidien LLC, Mansfield, MA, USA) immediately before commencement of corralling, and approximately every 30 min thereafter. Samples were placed in 5 mL of viral transport media^10^ and frozen until testing. Results of another study sought to determine IAV prevalence in the pig population by nasal wipe collection as pigs proceeded through the chute using previously described methods (The Ohio State University Animal Use Protocol 2009A0134-R2) and were used for comparison.^11^

All samples were tested in parallel for IAV with real-time reverse transcription-polymerase chain reaction (rRT-PCR) and virus isolation in Madin-Darby Canine Kidney (MDCK) cells; recovered isolates were subtyped using previously described protocols.^2^

In total, 236 environmental samples were collected at Fairs 1–6. IAV was detected via rRT-PCR in 26 (11.0%) environmental samples at two fairs, Fairs 1 and 2. Seven (3.0%) IAV isolates were recovered from environmental samples at Fairs 1 and 2. IAV-positive pigs were found at the same two fairs. IAV was not detected in the environment or pigs at the remaining four fairs. At Fair 1, 10 of 33 (30.3%) environmental samples tested positive via rRT-PCR for IAV and five (15.2%) isolates were recovered (Figure 1B). At Fair 2, 17 of 42 (40.5%) environmental samples tested positive via rRT-PCR for IAV and two (4.8%) isolates were recovered (Figure 1C). The nucleotide sequences for six of the seven IAV environmental isolates were obtained and are available on GenBank (Supplementary Table S1). Although no human cases were reported in association with these fairs, the genotypes of the recovered IAV isolates have been associated with variant influenza infections in humans.

Time of sample collection from the start of corralling (minutes post-start) was recorded (or estimated when data were missing) for environmental and individual pig samples. All environmental samples taken prior to the start of corralling tested negative for IAV. Comparing individual pig data shows that before environmental surfaces tested positive, several IAV-positive pigs with low cycle threshold (Ct) values at Fair 1 moved through the chute (135 min post-start; Figure 1A). Similarly, at Fair 2, virus was found on environmental surfaces immediately after the majority of IAV-positive pigs had moved through the chute (150 min post-start; Figure 1B). At both fairs, environmental subtypes matched those of the pigs with a mixture of H1N2 and H1/3N2 subtypes and an H3N2 subtype at Fairs 1 and 2, respectively.

While length of exhibition, direct contact between pigs, and large pig populations have been proposed as enhancing IAV transmission during agricultural fairs,^11^ this study provides insight into how corralling activities can potentially drive high IAV prevalence. The recovery of viable IAVs from environmental surfaces during corralling illustrates that this activity can increase virus transmission in exhibition swine. Previous testing found pigs sampled during movement through the chute have a higher IAV prevalence compared with when arriving pigs are sampled on trailers or in respective pens. As IAV-positive pigs move through the chute, nasal secretions are left behind on pig-contact surfaces allowing each subsequent pig to contact residual virus, thereby creating an indirect transmission pathway.^2^ Although a few swine samples were detected with high Ct values during the early part of corralling at both Fair 1 and Fair 2, once pigs with low Ct values (that is, truly positive and actively shedding virus) proceeded through the chute, virus was detected on environmental surfaces. Pigs moving through the same chute after these low Ct value pigs were more likely to test positive via rRT-PCR, albeit at high Ct values, than pigs moving through prior to the low Ct value pigs. It is presumed that most high Ct count positive pigs were not yet infected at time of sampling, but rather IAV was deposited on the pigs' snouts during transit through the chute and this initial inoculation was subsequently detected by the nasal wipe sampling. Exposure of naïve pigs to IAV during the entry process could explain subsequent infection, accelerated transmission and increase in IAV prevalence by the close of the fairs, five to seven days later.

With clear epidemiological links between IAV in swine at agricultural fairs and outbreaks in humans attending the same fairs,^5, 12^ an environment for IAV transmission is created by increased swine-human contact. Precautions must be taken to reduce IAV transmission and prevalence to protect public health. Limiting swine-human contact during agricultural fairs would likely decrease swine-to-human IAV transmission but would not impact the increase in IAV-positive swine. Show officials should use mitigation procedures, such as rinsing with water, cleaning, and wiping down environmental surfaces, where mechanical force is likely to reduce the amount of virus with which subsequent pigs may contact. Where rinsing fails or is not feasible, disinfecting chutes with animal-safe disinfectants will likely decrease viral burden on these surfaces significantly. Such cleaning and disinfecting procedures would be expected to decrease virus transmission between pigs upon fair entry, thereby decreasing IAV prevalence in swine during the fair, and ultimately reduce the public health threat.

This study demonstrates that swine-contact surfaces used during corralling are important fomites in indirect transmission of IAV. It also provides insight into the role of environmental surfaces in IAV transmission during and after swine exhibitions. With evidence of virus contamination of environmental surfaces, mitigation strategies targeting IAV control during this and similar processes is paramount.

## Figures and Tables

**Figure 1:**
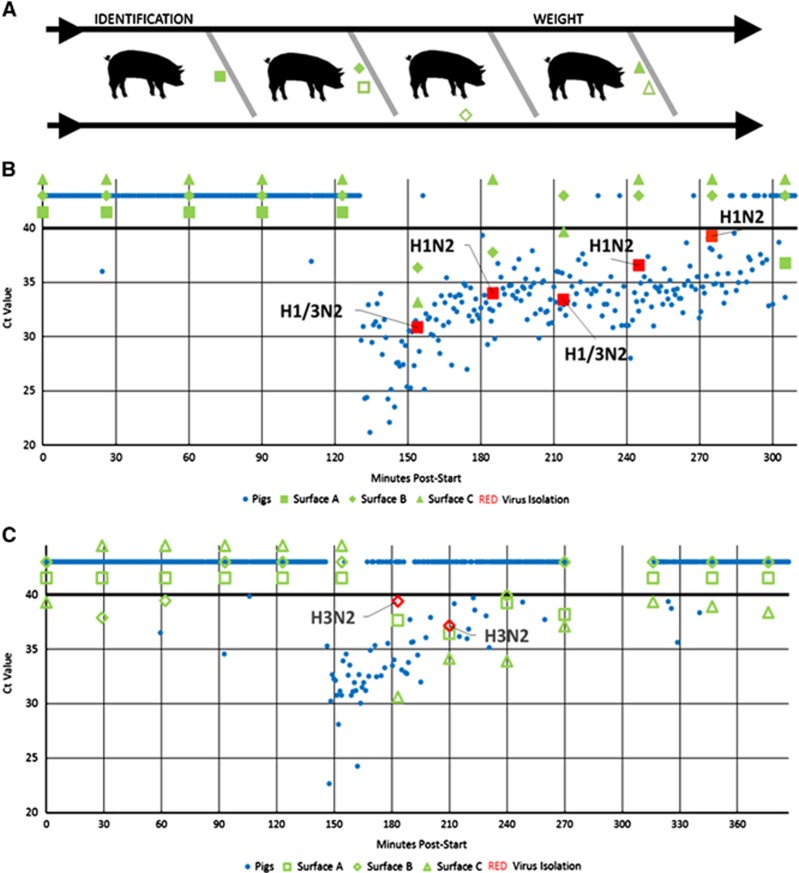
Chute diagram and corresponding rRT-PCR and virus isolation results. (**A**) Chute diagram. Black lines represent stationary side gates while diagonal gray bars represent moveable dividers that allow individual pigs to be held for procedures such as identification and weight determination. Arrows indicate direction of pig movement. Closed shapes represent surfaces sampled at Fair 1 and open shapes represent surfaces sampled at Fair 2. (**B**) and (**C**) Fair 1 and Fair 2, respectively, environmental rRT-PCR and virus isolation data compared with individual pig rRT-PCR data. The *x* axes represent number of minutes after commencement of corralling at which each sample was taken. The *y* axes represent rRT-PCR Ct values of each sample. Any Ct value under 40 is positive. Negative samples were assigned ambiguous Ct values and appear above 40. The color red denotes viral isolates. Subtypes are shown. real-time reverse transcription-polymerase chain reaction, rRT-PCR.
